# Sex Difference and Rupture Rate of Intracranial Aneurysms: An Individual Patient Data Meta-Analysis

**DOI:** 10.1161/STROKEAHA.121.035187

**Published:** 2022-01-05

**Authors:** Charlotte C.M. Zuurbier, Rob Molenberg, Liselore A. Mensing, Marieke J.H. Wermer, Seppo Juvela, Antti E. Lindgren, Juha E. Jääskeläinen, Timo Koivisto, Tomosato Yamazaki, Maarten Uyttenboogaart, J. Marc C. van Dijk, Marlien W. Aalbers, Akio Morita, Shinjiro Tominari, Hajime Arai, Kazuhiko Nozaki, Yuichi Murayama, Toshihiro Ishibashi, Hiroyuki Takao, Renato Gondar, Philippe Bijlenga, Gabriel J.E. Rinkel, Jacoba P. Greving, Ynte M. Ruigrok

**Affiliations:** UMC Utrecht Brain Center, Department of Neurology and Neurosurgery (C.C.M.Z, L.A.M., G.J.E.R., Y.M.R.), University Medical Center Utrecht, the Netherlands.; Julius Center for Health Sciences and Primary Care (J.P.G.), University Medical Center Utrecht, the Netherlands.; Departments of Neurosurgery (R.M., J.M.C.v.D., M.W.A.), University Medical Center Groningen, the Netherlands.; Neurology and Medical Imaging Center (M.U.), University Medical Center Groningen, the Netherlands.; Department of Neurology, Leiden University Medical Center, the Netherlands (M.J.H.W.).; Department of Clinical Neurosciences, University of Helsinki, Finland (S.J.).; Department of Clinical Radiology (A.E.L, J.E.J., T.K.), Kuopio University Hospital, Finland.; Neurosurgery of NeuroCenter (A.E.L, J.E.J., T.K.), Kuopio University Hospital, Finland.; Institute of Clinical Medicine, School of Medicine, Faculty of Health Sciences, University of Eastern Finland, Kuopio (A.E.L, J.E.J., T.K.).; Department of Neurosurgery, National Hospital Organization, Mito Medical Center, Japan (T.Y.).; Medical Center UCAS Japan Coordinating Office- University of Tokyo- Nippon Medical School, Neurological Surgery (A.M.).; Department of Health Informatics, School of Public Health, Kyoto University, Japan (S.T.).; Department of Neurosurgery, Juntendo University- Medical School, Tokyo, Japan (H.A.).; Department of Neurosurgery, Shiga University of Medical Science, Japan (K.N.).; Department of Endovascular Neurosurgery, Tokyo Jikei University School of Medicine, Japan (Y.M., T.I., H.T.).; Neurosurgery Division, Department of Clinical Neurosciences, Faculty of Medicine, Geneva University Medical Center, Switzerland (R.G., P.B.).

**Keywords:** intracranial aneurysm, prevalence, risk factor, sex, subarachnoid hemorrhage

## Abstract

**Background and Purpose:**

In previous studies, women had a higher risk of rupture of intracranial aneurysms than men, but female sex was not an independent risk factor. This may be explained by a higher prevalence of patient- or aneurysm-related risk factors for rupture in women than in men or by insufficient power of previous studies. We assessed sex differences in rupture rate taking into account other patient- and aneurysm-related risk factors for aneurysmal rupture.

**Methods:**

We searched Embase and Pubmed for articles published until December 1, 2020. Cohorts with available individual patient data were included in our meta-analysis. We compared rupture rates of women versus men using a Cox proportional hazard regression model adjusted for the PHASES score (Population, Hypertension, Age, Size of Aneurysm, Earlier Subarachnoid Hemorrhage From Another Aneurysm, Site of Aneurysm), smoking, and a positive family history of aneurysmal subarachnoid hemorrhage.

**Results:**

We pooled individual patient data from 9 cohorts totaling 9940 patients (6555 women, 66%) with 12 193 unruptured intracranial aneurysms, and 24 357 person-years follow-up. Rupture occurred in 163 women (rupture rate 1.04%/person-years [95% CI, 0.89–1.21]) and 63 men (rupture rate 0.74%/person-years [95% CI, 0.58–0.94]). Women were older (61.9 versus 59.5 years), were less often smokers (20% versus 44%), more often had internal carotid artery aneurysms (24% versus 17%), and larger sized aneurysms (≥7 mm, 24% versus 23%) than men. The unadjusted women-to-men hazard ratio was 1.43 (95% CI, 1.07–1.93) and the adjusted women/men ratio was 1.39 (95% CI, 1.02–1.90).

**Conclusions:**

Women have a higher risk of aneurysmal rupture than men and this sex difference is not explained by differences in patient- and aneurysm-related risk factors for aneurysmal rupture. Future studies should focus on the factors explaining the higher risk of aneurysmal rupture in women.

## Abstract 2

Supplemental Digital Content is available in the text.

Approximately 3% of the general population has an unruptured intracranial aneurysm (UIA).^1^ Rupture of an intracranial aneurysm results in aneurysmal subarachnoid hemorrhage (aSAH), a subtype of stroke which carries a high morbidity and case fatality.^2^ UIA and aSAH occur more often in women than in men with overall 65% of the patients being women.^1,3^

In the decision whether to treat UIA with neurosurgical or endovascular treatment to prevent future aSAH, the risk of rupture and the risk of complications of preventive treatment have to be balanced.^4^ The 5-year risk of rupture of UIA can be assessed using the PHASES score (Population, Hypertension, Age, Size of Aneurysm, Earlier Subarachnoid Hemorrhage From Another Aneurysm, Site of Aneurysm), which takes into account several patient- and aneurysm-related factors associated with rupture including geographic location, hypertension, age, history of aSAH, aneurysm size, and location.^5^ The PHASES score is based on a pooled analysis of individual patient data from prospective cohort studies on rupture rates of UIAs and risk factors for rupture. In this pooled analysis, women had a higher risk of rupture, but in multivariable analysis, female sex was not an independent risk factor. Another meta-analysis including both retrospective and prospective studies reported a statistically significantly higher rupture risk in women compared to men, but whether female sex was an independent risk factor could not be investigated because multivariable analysis was not possible due to lack of individual patient data.^6^ The higher risk of UIA rupture in women may therefore be explained by a higher prevalence of patient- or aneurysm-related risk factors for UIA rupture in women.

We performed a pooled analysis of individual patient data from prospective cohort studies to assess if sex is a risk factor for intracranial aneurysm rupture independent from other risk factors for rupture including the PHASES score, smoking, and a positive family history for aSAH.

## Methods

### Search Strategy and Selection Criteria

We performed a systematic search of the Pubmed and Embase database to retrieve all studies on rupture risk published up to December 1, 2020. We used the keywords “(intracranial aneurysm(s) OR cerebral aneurysm(s) AND (risk of rupture OR aneurysm rupture OR risk factors OR rupture OR unruptured OR subarachnoid hemorrhage) AND (follow up OR natural history OR natural course)” (Figure S1). In addition, we checked the reference list of all relevant publications for further eligible studies. We performed our systematic review and meta-analysis according to the Preferred Reporting Items for Systematic Reviews and Meta-Analyses recommendations and Meta-Analysis of Observational Studies in Epidemiology guidelines.^7,8^ We included studies that (1) used a prospective study design; (2) included at least 50 patients with UIA; and (3) studied the rupture rate of UIA and risk factors for aneurysm rupture. There was no language restriction other than the requirement of an abstract in English. When multiple publications reported on the same study population, the most recent publication was used. One author (C.C.M.Z.) performed the literature search, checked the titles and abstracts for studies meeting the inclusion criteria. Next, full-text copies of eligible studies were reviewed.

**Figure 1.**
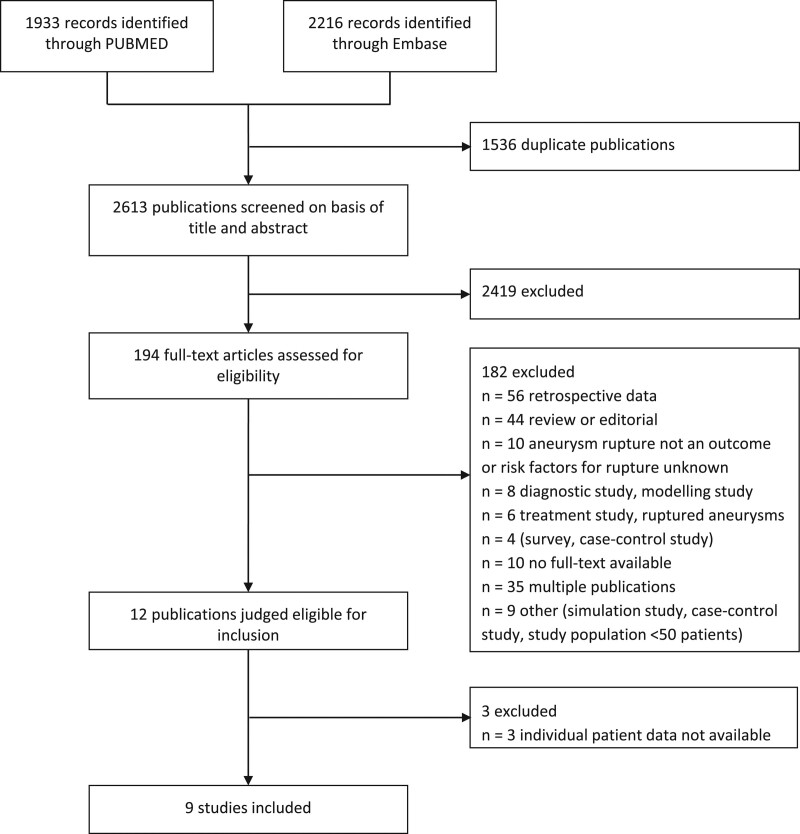
Preferred Reporting Items for Systematic Reviews and Meta-Analyses flow diagram.

In total, 2613 articles were screened (Figure 1). For the eligible studies meeting the inclusion criteria, we approached the research groups that performed these studies asking if they could provide us with their individual patient data. Only cohorts with available individual patient-level data were included in our meta-analysis. We found twelve studies that fulfilled the inclusion criteria,^9–19^ and 9 research groups provided us with their individual patient data.^12–19^ One of these population-based cohort studies on UIA, did not report on family history,^20^ but its authors could provide data including data on family history for aSAH for a selection of cases. These were data on patients with UIA collected between 1980 and 2017 from the IA database of Neurosurgery of Kuopio University Hospital and included 1181 patients with 1653 UIA, of whom 693 were women. The 9 cohorts are listed in Table 1, and the baseline characteristics of patients in all separate cohorts are listed in Table S1. Quality assessment of included cohort studies by QUIPS tool is shown in Table S2.

**Table 1.**
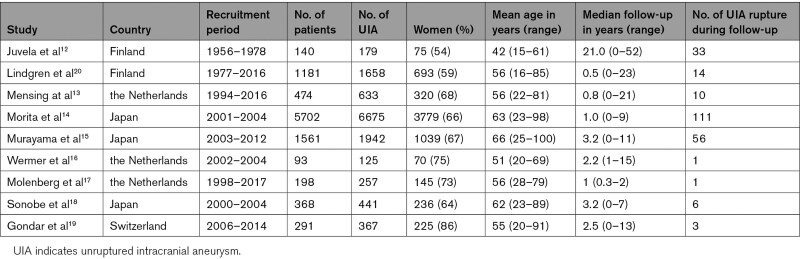
Baseline Characteristics of Included Studies

### Data Extraction

Data requested for each patient of the different included studies were the following: age, sex, history of aSAH, smoking status, positive family history for aSAH, hypertension status, number of aneurysms, maximum diameter of aneurysms, aneurysm location. These data were collected at baseline only and not at later time points. These data were recorded individually and also summarized in the PHASES score which includes data on the risk factors geographic location, hypertension, age, history of aSAH, aneurysm size, and location.^5^ Data requested for each patient during follow-up were the following: occurrence of rupture, date of rupture, data of a surgical or endovascular intervention, date of death, date of last follow-up assessment, and whether a patient was lost to follow-up. A smoker was defined as a former or current smoker, and person with hypertension as a systolic blood pressure >140 mm Hg or diastolic blood pressure >90 mm Hg or use of antihypertensive drugs. Individuals with a positive family history were defined as individuals with at least 2 affected first-degree relatives with aSAH whether or not in combination of first-degree relatives with UIA. The location of the aneurysm was classified as the internal carotid artery, posterior communicating artery, anterior cerebral arteries (including the anterior cerebral artery, anterior communicating artery, and pericallosal artery), middle cerebral artery, or posterior circulation (including the vertebral artery, basilar artery, cerebellar arteries, and posterior cerebral artery). Patients with polycystic kidney disease and moyamoya disease were excluded. We predefined the primary end point as the rupture of UIA.

### Statistical Approach

Missing data were imputed for smoking, hypertension, and family history of aSAH within each cohort using the linear regression method (multivariable analyses). To assign values for these missing data, we performed multiple imputation creating 10 imputation datasets using all relevant prognostic factors and outcome. A sensitivity analysis was done by excluding participants for whom data were missing. In one study only data on current smoking was available but no data on former smoking, and therefore, in our analysis data on current smoking was considered as current or former smoking.^17^ Fifty-seven Japanese patients were included both in the cohort of Morita et al^14^ and of Murayama et al,^15^ whereas 11 patients were included in both the cohort of Mensing et al^13^ and of Wermer et al.^16^ In the pooled analysis, these patients were removed from one of these cohorts. Categorical variables of baseline characteristics were compared using the χ^2^ test. Continuous variables of baseline characteristics were compared among groups using the Mann-Whitney *U* test or the Student *t* test. A p≤0.05 was considered statistically significant. We pooled the individual patient data of the included studies and estimated sex-specific rupture rates for each cohort separately. In case of multiple UIAs, the largest UIA was used to categorize the patient regarding site and size of the aneurysm. In addition, we performed an aneurysm-based analysis where all UIAs were analyzed. Rupture rate was analyzed with a per-patient analysis and a per aneurysm analysis using a Cox proportional hazard regression model, adjusted for the PHASES score,^5^ smoking, and positive family history for aSAH. A 2-stage approach was used with random effect for cohort because we expected heterogeneity since studies were performed in different countries which used different treatment regimes, and a fixed effect for the PHASES score, smoking, and positive family history for aSAH. As a sensitivity analysis, we also performed a one-stage model. Proportional hazard assumptions were checked in each individual cohort using diagnostics based on the scaled Schoenfeld residuals.^21^ Follow-up data for patients started at time of UIA diagnosis and patients were followed up until aneurysmal rupture occurred. Patients were censored at the time of death, last follow-up assessment, or at the time of surgical or endovascular aneurysm treatment without preceding rupture. When patients underwent a surgical or endovascular aneurysm treatment, data from the period up to the time of the intervention were included in the analysis, whereas data from the period after the intervention were not included. The data that support the findings of this study are available from the corresponding author upon reasonable request.

## Results

We pooled individual patient data from 9940 patients with 12 193 UIAs and 24 357 person-years follow-up using data from nine prospective cohort studies.^12–20^ Studies were at low and moderate risk of bias. Baseline characteristics of patients are shown in Table 2. Data on patient characteristics was almost complete except for smoking which was available in 9705/9940 (98%), for hypertension which was available in 9853/9940 (99%), and for family history of aSAH which was available in 9794/9940 (99%). Information on outcome measure was complete for all patients. The mean age was 61±12 years, 6555 patients (66%) were women, and patients came from Dutch (8%), Finnish (12%), Japanese (77%), and Swiss (4%) populations. Women were older (61.9 versus 59.5 years), less often smokers (20% versus 44%), and more often had internal carotid artery aneurysms (24% versus 17%) and larger aneurysms (≥7 mm, 24% versus 23%) than men. There were more women than men in cohorts from Japan (67% versus 33%), the Netherlands (70% versus 30%), Switzerland (77% versus 23%), whereas this difference was less pronounced in the cohort from Finland (58% versus 42%). The median PHASES score was the same in women (7.0 [range, 0–21]) and men (7.0 [range, 0–20]), and the mean PHASES score was 7.2±3.2 in women and 7.4±3.0 in men. In our pooled analysis, the mean follow-up time for women was 2.4±3.5 years (median: 1.5 (0-52) year) and 2.5±3.7 years (median: 1.5 (0-50) year) for men. Preventive neurosurgical or endovascular treatment during follow-up occurred in 36% of women (median: 60 days) and in 37% of men (median: 61 days). When assessing these characteristics per UIA, similar differences in characteristics were found (data not shown).

**Table 2.**
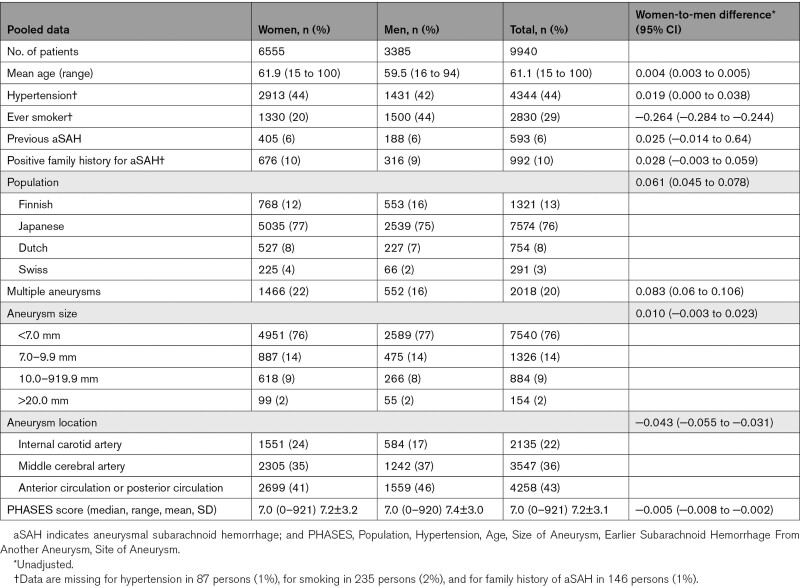
Baseline Characteristics of Included Patients

In 234 patients, rupture of the single, largest or another than the largest UIA occurred. Of these 234 patients, 67 patients had multiple UIA, and in 226 of 234 patients (97%), the single aneurysm (n=167) or the largest aneurysm in case of multiple aneurysms (n=59) ruptured. In 8 of the 67 patients with multiple aneurysm, another than the largest aneurysm ruptured. Of the 226 patients in whom the single or largest UIA ruptured, 163 were women (rupture rate 1.04%/person-years [95% CI, 0.89–1.21]), and 63 men (0.74%/person-years [95% CI, 0.58–0.94]). Characteristics of ruptured aneurysms are shown in Table 3.

**Table 3.**
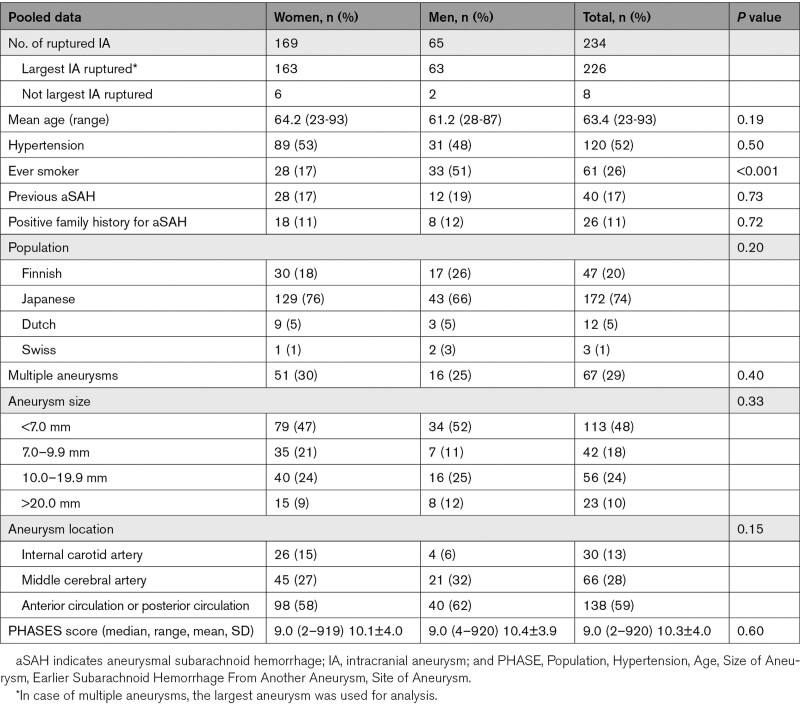
Characteristics of Ruptured Intracranial Aneurysms, per Aneurysm

**Figure 2.**
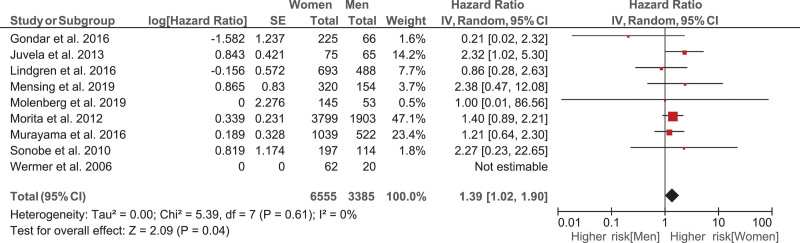
Hazard ratio of the rupture rate in women compared to men adjusted for the PHASES score (Population, Hypertension, Age, Size of Aneurysm, Earlier Subarachnoid Hemorrhage From Another Aneurysm, Site of Aneurysm), smoking and positive family history of aneurysmal subarachnoid hemorrhage, analyzing the data per patient.

**Figure 3.**
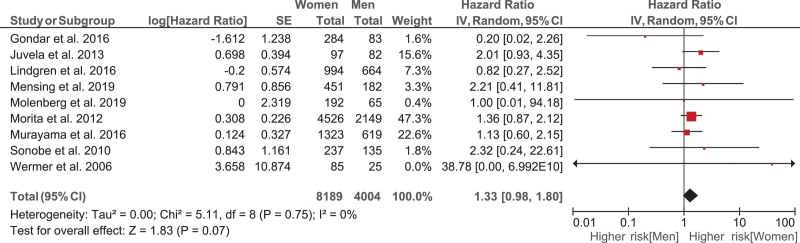
Hazard ratio of the rupture rate in women compared to men adjusted for the PHASES score (Population, Hypertension, Age, Size of Aneurysm, Earlier Subarachnoid Hemorrhage From Another Aneurysm, Site of Aneurysm), smoking and positive family history of aneurysmal subarachnoid hemorrhage, analyzing the data per aneurysm.

The unadjusted women-to-men hazard ratio was 1.43 (95% CI, 1.07–1.93). After adjustment for the PHASES score, smoking, and positive family history for aSAH, the women-to-men hazard ratio was slightly lower (1.39 [95% CI, 1.02–1.90]; Figure 2). In the sensitivity analysis on the subset of patients with no missing data for smoking, hypertension, and family history of aSAH (n=9566), we found similar but nonstatistically significant results (Figure S2). We also performed a one-stage model which resulted in a hazard ratio of 1.36 (95% CI, 1.01–1.85). In the aneurysm-based analysis where all UIAs were analyzed, the results were essentially the same (Figure 3).

## Discussion

In our pooled analysis of individual patient data from prospective cohort studies, we found that women have a higher risk of aneurysmal rupture, and this increased rupture risk for women is not explained by differences in patient- and aneurysm-related risk factors for aneurysmal rupture, being risk factors of the PHASES score, smoking, and a positive family history for aSAH.

Some of the risk factors for rupture were more often present in women, but others in men. As the patient- and aneurysm-related risk factors for which we corrected in our analysis, do not explain the increased rupture risk in women, additional factors contributing to the increased risk remain to be detected. We had no data on the shape of the aneurysm in our data set. Because aspect ratio and irregular aneurysm shape are also known factors for UIA rupture,^22,23^ a higher prevalence of irregular aneurysms in women than in men may contribute to the sex difference in rupture, but it is unlikely that such a difference would explain the sex difference in rupture completely. Because we could not find data in the literature on sex differences regarding shape of the aneurysms, it is currently unknown if or to what extent differences in shape of aneurysms between women and men play a role in the higher rupture risk in women.

Additional factors explaining the sex difference in risk of UIA rupture may be female-specific hormonal and reproductive factors. A previous systematic literature review on female risk factors for aSAH found an increased risk of aSAH for postmenopausal versus premenopausal women although the pathophysiology of this effect and its influence on the difference in incidence of SAH between the sexes remains unclear.^24^ Alternatively, female-specific genetic factors, such as genetic factors of the X-chromosome, sex-specific effects of environmental risk factors, such as smoking^25^ or other yet unknown clinical factors which occur more often or have stronger effect in women than in men may explain the difference.

Our study has several strengths. It includes a large data set with individual patient data from several cohorts including risk factors for aneurysmal rupture. Also, almost all study cohorts included in this meta-analysis showed a higher rupture rate in women compared to men. This means that our data are consistent and generalizable for both Asian and European countries.

A first limitation of this study is that selection bias may have occurred due to informative censoring (loss to follow-up) within each cohort study. If men were treated more aggressively during follow-up than women for example upon growth of the UIA, which is associated with a higher risk of rupture,^26^ this may have led to selection bias. However, we found no difference in preventive neurosurgical or endovascular treatment during follow-up between men and women as it was done in 36% of women (median: 60 days) and in 37% of men (median: 61 days). Therefore, it is unlikely that differences in preventive treatment have influenced our results considerably. Second, in most studies, we only had data on smoking at the time of UIA detection but not for smoking status during follow-up. As a previous study showed that continuation of smoking is a significant risk factor for UIA rupture, no conclusions can be drawn about the effect of a change in smoking status after aneurysm detection during follow-up on our outcomes.^27^ Cessation of smoking might have occurred more often in men during follow-up compared to women. Similarly, in most studies, we only had data on hypertension at time of UIA detection and not during follow-up. Better control of blood pressure might have been achieved in men during follow-up compared with women. Third, although 9 research groups^12–20^ provided us with their individual patient data, 3 research groups^9–11^ were not able to do so, which could possibly lead to a bias. However, the population characteristics between the three cohorts not included (Matsumoto et al^9^: 63% female, Güresir et al^10^: 78% female, and ISUIA [International Study of Unruptured Intracranial Aneurysms Investigators]^11^: 75% female) and rupture risk (Matsumoto et al^9^: 6/111 patients, all female; Güresir et al^10^: 3/263 patients, all female; and ISUIA^11^: 51/1692, sex unknown) differed not much from those of the nine cohorts analyzed (66% [range, 54–86] female), and therefore, do not think that such a potential bias influences our conclusions. Fourth, in our analysis, patients from Japanese populations were overrepresented (77%) compared to Dutch (8%), Finnish (12%), and Swiss (4%) populations. Except for a small study in the Swiss population, in all populations, a higher risk of rupture for women compared to men was found, so we think our results are generalizable to all populations. Fifth, in our study, we performed patient-level analysis, and in patients with multiple UIAs, we analyzed data of the largest UIA, which is not always the UIA that actually ruptures.^28^ However, in our analysis for rupture rate on aneurysm level, we found comparable results.

## Conclusions

Our results show that UIAs in women have a higher rupture risk than UIAs in men, which is not explained by differences in patient- and aneurysm-related risk factors for aneurysmal rupture, being risk factors of the PHASES score, smoking, and a positive family history for aSAH. When assessing the risk of rupture of UIAs in women, this higher risk should be taken into account and a more aggressive treatment approach in women as compared to men is justified. Future studies should focus on the identification of the factors explaining the higher rupture risk of UIA in women, such as different approach during follow-up, female-specific hormonal and reproductive factors, or female-specific genetic and environmental risk factors.

## Article Information

### Acknowledgments

Drs Mensing, Molenberg, van Dijk, Aalbers, Wermer, Juvela, Lindgren, Jääskeläinen, Koivisto, Yamazaki, Uyttenboogaart, Morita, Tominari, Arai, Nozaki, Murayama, Ishibashi, Takao, Gondar, Bijlenga, Rinkel, Greving and Ruigrok contributed to data collection. Dr Zuurbier searched the literature. Dr Rinkel, Ruigrok, Greving, and Zuurbier communicated with coauthors for individual patient data. Dr Zuurbier performed the data analysis under the supervision of Dr Ruigrok, Greving, and Rinkel. Dr Zuurbier generated the figures. Dr Zuurbier wrote the first draft of the article and all authors revised the article critically and approved the final version.

### Sources of Funding

We acknowledge the support from the Netherlands Cardiovascular Research Initiative: An initiative with support of the Dutch Heart Foundation, CVON2015-08 ERASE (Optimal Early Recognition of Persons at High Risk of Aneurysmal Subarachnoid Hemorrhage). This project has received funding from the European Research Council (ERC) under the European Union’s Horizon 2020 research and innovation program (grant agreement No. 852173). The Swiss study was performed within the framework of the AneuX project supported by SystemsX.ch, and evaluated by the Swiss National Science Foundation (2014/261).

### Disclosures

None.

### Supplemental Material

Figure S1

Table S1–S2

## Supplementary Material


