# Supplementary material for: Sex Difference and Rupture Rate of Intracranial Aneurysms: An Individual Patient Data Meta-Analysis
Source: Stroke. 2022 Jan 5;53(2):362–9. doi: 10.1161/STROKEAHA.121.035187 (PMC8785514; doi:10.1161/STROKEAHA.121.035187)

SUPPLEMENTAL MATERIAL

Supplemental Table 1. Baseline characteristics of all separate cohorts.

Supplemental Table II. Risk of bias assessment tool (QUIPS).

Supplemental Table 111. Baseline characteristics of all patients with and without aneurysm rupture.

Supplemental Figures I. Search strings.

Supplemental Figure II. Complete case analysis: hazard ratio (HR) of the rupture rate in women compared to men adjusted for the PHASES score, smoking and positive family history of aneurysmal subarachnoid hemorrhage, analyzing the data per patient.

Supplemental Table I. Baseline characteristics of all separate cohorts.

	Juvela et al[12]		Lindgren et al[20]		Mensing et al[13]		Wermer et al[16]		Molenberg et al[17]		Sonobe et al[18]		Morita et al[14]		Murayama et al[15]		Gondar et al[19]	
	Women	Men	Women	Men	Women	Men	Women	Men	Women	Men	Women	Men	Women	Men	Women	Men	Women	Men
Number of patients	75	65	693	488	320	154	70	23	145	53	236	132	3799	1903	1039	522	225	66
Age (mean, SD)	42 ± 11	41 ± 10	57 ± 13	54 ± 11	55 ± 11	57 ± 11	51 ± 10	51 ± 11	56 ± 11	57 ± 11	63 ± 10	60 ± 10	63 ± 10	61 ± 10	66 ± 12	64 ± 11	56 ± 15	53 ± 15
Hypertension	24 (32)	26 (40)	302 (44)	186 (38)	130 (41)	67 (44)	38 (54)	11 (48)	70 (48)	19 (36)	107 (45)	53 (40)	1682 (44)	789 (41)	479 (46)	253 (49)	101 (45)	38 (58)
Smoking	39 (52)	60 (92)	273 (39)	310 (64)	146 (46)	61 (40)	62 (89)	22 (96)	76 (52)	29 (55)	89 (38)	85 (64)	340 (9)	617 (32)	220 (21)	287 (55)	112 (50)	41 (62)
Previous aSAH	67 (89)	62 (95)	21 (3)	23 (5)	0	0	59 (84)	18 (78)	68 (47)	13 (25)	25 (11)	9 (7)	138 (4)	48 (3)	28 (3)	15 (3)	3 (1)	0
Positive family history	12 (16)	15 (23)	161 (23)	87 (18)	44 (14)	18 (12)	23 (33)	6 (26)	42 (29)	18 (34)	24 (10)	12 ((9)	220 (6)	107 (6)	134 (13)	50 (10)	27 (12)	10 (15)
Multiple aneurysms	19 (25)	14 (22)	247 (36)	150 (31)	86 (27)	23 (15)	19 (27)	5 (22)	7 (5)	8 (15)	42 (18)	23 (17)	587 (15)	201 (11)	387 (37)	121 (23)	47 (21)	9 (14)
Size																		
< 7.0 mm	61 (81)	54 (83)	484 (70)	332 (68)	154 (48)	67 (44)	70 (100)	23 (100)	127 (88)	37 (70)	236 (100)	132 (100)	2670 (70)	1402(74)	984 (95)	505 (97)	212 (94)	59 (89)
7.0-9.9 mm	8 (11)	8 (12)	97 (14)	88 (18)	85 (27)	47 (31)	0	0	14 (10)	10 (19)	0	0	651 (17)	306 (16)	24 (2)	10 (2)	8 (4)	5 (8)
10.0-19.9 mm	4 (5)	2 (3)	92 (13)	49 (10)	69 (22)	32 (21)	0	0	4 (3)	4 (8)	0	0	418 (11)	172 (9)	25 (2)	5 (1)	5 (2)	2 (3)
> 20.0 mm	2 (3)	1 (2)	20 (3)	19 (4)	11 (3)	8 (5)	0	0	0	2 (4)	0	0	60 (2)	23 (1)	6 (1)	2 (0)	0	0
Aneurysm location																		
ICA	37 (49)	23 (35)	140 (20)	66 (14)	63 (20)	10 (7)	11 (16)	2 (9)	29 (20)	9 (17)	92 (39)	50 (38)	789 (21)	282 (15)	311 (30)	125 (24)	90 (40)	20 (30)
MCA	30 (40)	32 (49)	321 (46)	230 (47)	106 (33)	68 (44)	29 (41)	10 (44)	62 (43)	23 (43)	82 (35)	49 (37)	1333 (35)	694 (36)	281 (27)	128 (25)	80 (36)	18 (27)
ACA & P	8 (11)	10 (15)	232 (34)	192 (39)	151 (47)	76 (49)	30 (43)	11 (48)	54 (37)	21 (40)	62 (26)	33 (25)	1677 (44)	927 (49)	447 (43)	269 (52)	54 (24)	28 (42)

Phases (median, range)	8 (5-15)	8 (5-14)	9 (5-21)	9 (5-20)	5 (0-15)	5 (0-15)	4 (1-6)	5 (1-6)	4 (0-11)	4 (0-16)	6 (3-10)	5 (3-9)	7 (3-19)	7 (3-19)	7 (3-19)	7 (3-17)	2 (0-12)	4 (0-10)
Phases (mean, SD)	8.3 ± 2.4	8.6 ± 2.2	9.4 ± 3.2	9.6 ± 3.1	5.5 ± 3.3	6.1 ± 3.4	4.0 ± 1.6	4.2 ± 1.7	3.9 ± 2.0	4.6 ± 3.0	5.6 ± 1.8	5.4 ± 1.6	7.6 ± 3.1	7.5 ± 2.8	6.5 ± 2.5	6.5 ± 2.2	2.5 ± 2.1	3.4 ± 2.3
Ruptured aneurysms	22	10	8	6	8	2	0	0	0	1	5	1	79	27	41	14	1	2
Person-years of follow-up	1764.4	1259.2	1163.6	898.09	799.5	377.08	234.1	82.4	150.7	56.6	814.1	485.9	6329.77	3271.89	3858.28	1901.12	706.58	227.3
Follow-up years (median)	23.5 (1-52)	15.4 (1-50)	0.5 (0-18)	0.5 (0-23)	0.7 (0-21)	1 (0-18)	2.2 (1-15)	2.3 (1-10)	1 (0-2)	1 (0-2)	3.2 (0-7)	3.3 (0-7)	1.0 (0-8)	1.0 (0-9)	3.2 (0-11)	3.1 (0-11)	2.5 (0-13)	2.7 (0-10)
Rupture rate	1.25 (0.8-1.9)	0.79 (4.0-14.2)	0.69 (0.32-1.31)	0.69 (0.27-1.39)	1.0 (0.46-1.9)	0.53 (0.09-1.75)	-	-	-	1.77 (0.09-8.71)	0.61 (0.23-1.36)	0.21 (0.01-1.02)	1.25 (0.99-1.55)	0.83 (0.55-1.18)	1.06 (0.77-1.43)	0.74 (0.42-1.21)	0.14 (0.01-0.70)	0.88 (0.15-2.91)

aSAH: aneurysmal subarachnoid hemorrhage, SD: standard deviation, ICA: internal carotid artery, MCA: middle cerebral artery, ACA: anterior cerebral arteries, P: posterior circulation.

Supplemental Table II. Risk of bias assessment tool (QUIPS).

	Study participation	Study attrition	Prognostic factor measurement	Outcome measurement	Study confounding	Statistical analysis reporting
Juvela et al[12]	moderate	low	low	moderate	moderate	low
Mensing et al[13]	low	high	low	moderate	moderate	low
Morita et al[14]	low	moderate	low	moderate	moderate	low
Murayama at al[15]	low	low	low	low	moderate	low
Wermer et al[16]	low	low	low	moderate	low	low
Molenberg et al[17]	low	high	low	low	low	low
Sonobe et al[18]	low	moderate	low	low	low	low
Gondar et al[19]	low	low	low	moderate	low	low
Lindgren et al[20]	low	moderate	low	moderate	moderate	low

Table III. Baseline characteristics of patients with and without aneurysm rupture.

Pooled data	No aneurysm rupture (n,%)	Aneurysm rupture (n,%)	Total (n,%)	p-value
Number of patients	9714	226	9940	
Women	6392 (66)	163 (72)	6555 (66)	0.047
Mean age (range)	61.0 (15-100)	63.7 (23-93)	61.1 (15-100)	0.001
Hypertension #	4230 (44)	114 (50)	4344 (44)	0.039
Ever smoker #	2772 (29)	58 (26)	2830 (29)	0.34
Previous aSAH	556 (6)	37 (16)	593 (6)	<0.001
Positive family history for aSAH #	969 (10)	23 (10)	992 (10)	0.92
Population				0.004
Finnish	1275 (13)	46 (20)	1321 (13)	
Japanese	7408 (76)	166 (74)	7574 (76)	
Dutch	743 (8)	11 (5)	754 (8)	
Swiss	228 (3)	3 (1)	291 (3)	
Multiple aneurysms	1960 ()	58 ()	2018 (20)	<0.001

Aneurysm size				<0.001
< 7.0 mm	7435 (77)	105 (47)	7540 (76)	
7.0-9.9 mm	1320 (14)	42 (19)	1326 (14)	
10.0-19.9 mm	828 (9)	56 (25)	884 (9)	
> 20.0 mm	131 (1)	23 (10)	154 (2)	
Aneurysm location				<0.001
Internal carotid artery	2107 (22)	28 (12)	2135 (22)	
Middle cerebral artery	3483 (36)	64 (28)	3547 (36)	
Anterior circulation or posterior circulation	4124 (43)	134 (59)	4258 (43)	
Phases score (median, range, mean, standard deviation)	7.0 (0-21)<br>7.2 ± 3.1	9.0 (2-20)<br>9.8 ± 4.0	7.0 (0-21)<br>7.2 ± 3.1	<0.001

aSAH; aneurysmal subarachnoid hemorrhage.

Data are missing for hypertension in 87 persons (1%), for smoking in 235 persons (2%) and for family history of aSAH in 146 persons (1%).

Figure I. Search strings.

Pubmed search string
#1:
"intracranial aneurysm"[Title/Abstract] OR "intracranial saccular aneurysm"[Title/Abstract] OR
"cerebral aneurysm"[Title/Abstract] OR "intracranial aneurysms"[Title/Abstract] OR "intracranial
saccular aneurysms"[Title/Abstract] OR "cerebral aneurysms"[Title/Abstract]
#2:
"risk of rupture"[Title/Abstract] OR "aneurysm rupture"[Title/Abstract] OR "risk factors"[Title/Abstract]
OR "rupture"[Title/Abstract] OR "unruptured"[Title/Abstract] OR "subarachnoid hemorrhage"[Title/Abstract]
#3:
"follow-up"[Title/Abstract] OR "follow up"[Title/Abstract] OR "natural history"[Title/Abstract] OR "natural
course"[Title/Abstract]
#1 AND **#2** AND **#3**

Embase search string
#1:
'intracranial aneurysm':ti:ab OR 'intracranial saccular aneurysm':ti:ab OR 'cerebral aneurysm':ti:ab OR
'intracranial aneurysms':ti:ab OR 'intracranial saccular aneurysms':ti:ab OR 'cerebral aneurysms':ti:ab
#2:
'risk of rupture':ti:ab OR 'aneurysm rupture':ti:ab OR 'risk factors':ti:ab OR 'rupture':ti:ab OR
'unruptured':ti:ab OR 'subarachnoid hemorrhage':ti:ab – 339.423
#3:
'follow-up':ti:ab OR 'follow up':ti:ab OR 'natural history':ti:ab OR 'natural course':ti:ab - 688.714
#1 AND **#2** AND **#3**

Supplemental Table II. Complete case analysis: hazard ratio (HR) of the rupture rate in women compared to men adjusted for the PHASES score, smoking and positive family history of aneurysmal subarachnoid hemorrhage, analyzing the data per patient.

Study or Subgroup	log[Hazard Ratio]	SE	Women Total	Men Total	Weight	Hazard Ratio IV, Random, 95% CI
Gondar et al. 2016	-1.582	1.237	225	66	1.8%	0.21 [0.02, 2.32]
Juvela et al. 2013	-0.159	0.52	51	41	10.2%	0.85 [0.31, 2.36]
Lindgren et al. 2016	-0.266	0.592	667	481	7.9%	0.77 [0.24, 2.45]
Mensing et al. 2019	1.447	1.121	279	140	2.2%	4.25 [0.47, 38.25]
Molenberg et al. 2019	0	2.273	83	36	0.5%	1.00 [0.01, 86.06]
Morita et al. 2012	0.339	0.231	3799	1903	51.7%	1.40 [0.89, 2.21]
Murayama et al. 2016	0.189	0.328	1039	522	25.7%	1.21 [0.64, 2.30]
Sonobe et al. 2010	11.565	686	95	58	0.0%	105345.43 [0.00, Not estimable]
Wermer et al. 2006	0	0	61	20		Not estimable
**Total (95% CI)**			**6299**	**3267**	**100.0%**	**1.21 [0.87, 1.67]**

Heterogeneity: Tau² = 0.00; Chi² = 4.78, df = 7 (P = 0.69); I² = 0%
Test for overall effect: Z = 1.14 (P = 0.25)

Hazard Ratio
IV, Random, 95% CI

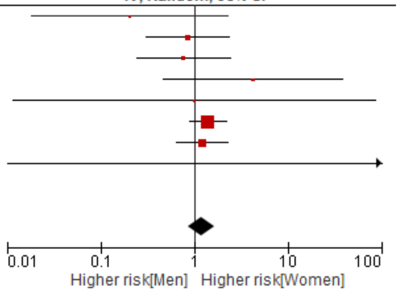

Supplement: Supplementary file 1 [file str-53-362-s001.pdf]
