# Supplementary figures and images for: Sex Difference and Rupture Rate of Intracranial Aneurysms: An Individual Patient Data Meta-Analysis
Source: Stroke. 2022 Jan 5;53(2):362–9. doi: 10.1161/STROKEAHA.121.035187 (PMC8785514; doi:10.1161/STROKEAHA.121.035187)

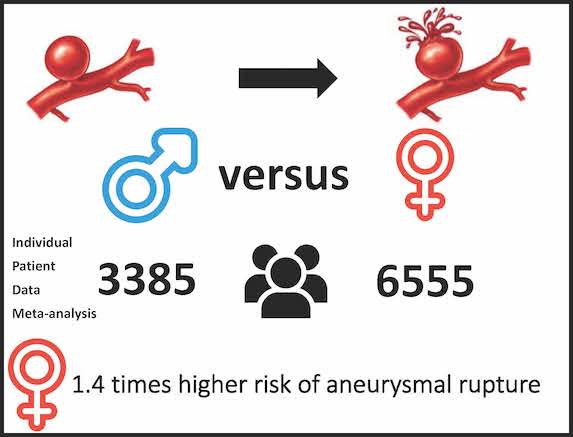

Supplement: Supplementary file 2 [file str-53-362-s002.jpg]
